# Associations of Assisted Reproductive Technology and Twin Pregnancy With Risk of Congenital Heart Defects

**DOI:** 10.1001/jamapediatrics.2019.6096

**Published:** 2020-02-24

**Authors:** Shi Wu Wen, Qun Miao, Monica Taljaard, Jane Lougheed, Laura Gaudet, Michael Davies, Andrea Lanes, Art Leader, Daniel J. Corsi, Ann E. Sprague, Mark Walker

**Affiliations:** 1 OMNI Research Group, Ottawa Hospital Research Institute, Ottawa, Ontario, Canada; 2 Department of Obstetrics and Gynecology, University of Ottawa Faculty of Medicine, Ottawa, Ontario, Canada; 3 School of Epidemiology and Public Health, University of Ottawa Faculty of Medicine, Ottawa, Ontario, Canada; 4 Nanhai Hospital, Southern Medical University, Foshan, Guangdong, China; 5 Better Outcomes Registry & Network (BORN) Ontario, Children’s Hospital of Eastern Ontario, Ottawa, Ontario, Canada; 6 Children’s Hospital of Eastern Ontario Research Institute, Ottawa, Ontario, Canada; 7 Division of Cardiology, Children’s Hospital of Eastern Ontario, Ottawa, Ontario, Canada; 8 Department of Pediatrics, University of Ottawa Faculty of Medicine, Ottawa, Ontario, Canada; 9 Robinson Research Institute, University of Adelaide, Adelaide, South Australia, Australia

## Abstract

**Importance:**

The extent to which assisted reproductive technology is associated with increased risk of congenital heart defects independent of its known association with twinning remains uncertain.

**Objective:**

To assess the extent to which assisted pregnancy is associated with increased risk of congenital heart defects independent of its known association with twinning.

**Design, Setting, and Participants:**

This retrospective cohort study linked records of congenital heart defect diagnoses with assisted reproductive technology cycles in 507 390 singleton or twin pregnancies (10 149 assisted pregnancies and 497 241 nonassisted pregnancies), including singleton and twin early pregnancy losses, stillbirths, and live births (follow-up to 1 year of age) in Ontario, Canada, between April 1, 2012, and October 31, 2015. Statistical analysis was performed from January 1, 2017, to September 9, 2019.

**Exposures:**

Assisted reproductive technology and its 2 subtypes: intracytoplasmic sperm injection and in vitro fertilization without intracytoplasmic sperm injection.

**Main Outcomes and Measures:**

The main outcome was congenital heart defects (prevalence and relative risk measured as odds ratios [ORs]). Mediation analysis was performed to assess the extent to which the association between assisted reproductive technology and congenital heart defects was mediated by twinning.

**Results:**

Of 507 390 mother-infant pairs with singleton or twin pregnancies evaluated, the prevalence of congenital heart defects in assisted pregnancies (223 [2.2%]) was higher than that in nonassisted pregnancies (6057 [1.2%]; crude OR, 1.82; 95% CI, 1.59-2.09). The strength of the association between assisted pregnancy and congenital heart defects decreased after adjusting for several risk factors simultaneously (adjusted OR, 1.70; 95% CI, 1.48-1.95). Further mediation analysis indicated that most of the association between assisted pregnancy and congenital heart defects was mediated by twinning (adjusted OR, 1.68; 95% CI, 1.44-1.92), and the natural direct association of assisted pregnancy with congenital heart defects among singleton pregnancies was 1.09 (95% CI, 0.93-1.25). Mediation of twinning accounted for 87.3% of the association.

**Conclusions and Relevance:**

Our study results suggest that the association between assisted reproductive technology and congenital heart defects may be mediated by twinning.

## Key Points

**Question:**

Is assisted reproductive technology associated with increased risk of congenital heart defects independent of its association with twin pregnancies?

**Findings:**

In this cohort study of 507 390 singleton or twin pregnancies, the prevalence of congenital heart defects was higher in assisted pregnancies (223 [2.2%]) than in nonassisted pregnancies (6057 [1.2%]). Twinning mediated 87.3% of the association between assisted pregnancy and congenital heart defects.

**Meaning:**

The findings suggest the association between assisted reproductive technology and congenital heart defects may be mediated by twinning.

## Teaser

This cohort study assesses the extent of association of assisted reproductive technology with congenital heart defects independent of its known association with twin pregnancies.

## Introduction

At present, approximately 2% of pregnancies are conceived by using assisted reproductive technology (ART), with upward trends in recent years.^1,2^ An increased risk of adverse pregnancy outcomes, including congenital heart defects (CHD), in ART pregnancies compared with non-ART pregnancies has been observed.^1,2,3,4,5,6,7,8,9,10,11,12,13,14,15^ Several maternal factors, such as maternal age, obesity, socioeconomic status, and preexisting health problems, have been associated with risks of both adverse pregnancy outcomes, including CHD and infertility requiring ART to conceive,^1,2,16,17,18^ and the association between ART and CHD tends to be reduced after adjustment for these maternal factors.^3,4,5,6,7,8,9,10,11,12,13,14,15^

One important consequence of increased use of ART is a substantially increased rate of twin pregnancies.^1,2,19,20,21,22,23,24,25,26^ Compared with singletons, the risks of adverse pregnancy outcomes including CHD have been shown to be increased in twins.^21,22,24,25,26^ Some studies have suggested that part of the increased risk of CHD observed in ART pregnancies may be attributable to the increased number of twin pregnancies.^10,14,26^

Congenital heart defect is a serious disease burden not only in infants but also in adolescents and adults.^27,28,29^ The pathophysiologic findings and pathogenesis of CHD are complicated and often are very different from other congenital anomalies.^12,13,30,31^ The American Institute of Ultrasound in Medicine, in collaboration with other professional organizations, has developed the Fetal Echocardiography Practice Parameter,^32^ and the American College of Radiology has endorsed this document. The association of ART and CHD should be further examined to decide whether fetal echocardiography, a costly procedure, should be used in ART pregnancies.^32^

We performed a large population-based study to examine the association between ART and CHD and to explore the associations of ART, twin pregnancies, and CHD, with twin status considered as a mediator in the analysis.

## Methods

### Study Design and Study Population

For this cohort study, ethical approval was obtained from the Ottawa Hospital Research Ethical Board, Ottawa, Ontario, Canada. Because this study used data collected by the province-approved registry with no additional data collection from patients, no informed consent was required. All singleton and twin stillbirths and live births (with follow-up to 1 year of age) and some early pregnancy losses that occurred between April 1, 2012, and October 31, 2015, in Ontario, Canada, and were captured by Better Outcomes Registry & Network (BORN) were included in the study. BORN is a provincial maternal child registry aiming to facilitate and improve care for mothers and children in Ontario.^33^ BORN has an internet-based data entry system administered by the Children’s Hospital of Eastern Ontario called the BORN Information System. There are 3 locations and periods in the BORN Information System in which CHD data can be captured: (1) prenatal screening records, (2) antenatal specialty clinic records, and (3) hospital records for birth or postpartum care or neonatal intensive care unit admissions. BORN has 100% capture for hospital and home births in Ontario. This database contains maternal demographic characteristics and health behaviors, preexisting maternal health problems, and birth outcomes. The national Canadian Assisted Reproductive Technologies Registry (CARTR Plus) is housed within BORN and is owned and administered by the medical directors of in vitro fertilization clinics and the Canadian Fertility and Andrology Society. The national CARTR Plus includes in vitro fertilization cycles for individuals undergoing ART-associated treatment in Canada.

### Outcome

Congenital heart defect was the outcome of interest in this study. To reduce misclassifications, only major structural CHD was considered (for a list of major structural CHD, see eTable 1 in the Supplement). Newborn diagnoses including CHD were collected at birth and during the postpartum period. Information on congenital anomalies in lost or terminated pregnancies was captured from prenatal screening follow-up visits if the anomaly was diagnosed through prenatal screening and antenatal specialty clinics if the anomaly was diagnosed through an antenatal clinic in Ontario. For CHD cases that were not captured by BORN, an additional ascertainment occurred through linkage with the Canadian Institute for Health Information (CIHI) Discharge Abstract Database and National Ambulatory Care Reporting System metadata. In CIHI records, CHD cases were identified based on a standardized diagnostic coding system, *International Classification of Diseases and Related Health Problems, Tenth Revision, Canada* (*ICD-10-CA*). We were able to capture CHD at birth and up to 1 year through linkage with CIHI records. Because of difficulties in separately identifying in utero fetal CHD cases in twins and limited further linking to infant records, we used individual pregnancy as the unit of analysis (ie, if 1 member of a twin pair was affected, that pregnancy was considered to be affected).

### Exposure

Assisted reproductive technology was the exposure of interest in the study. We followed the Centers for Disease Control and Prevention definition for ART,^34^ which included both intracytoplasmic sperm injection (ICSI) and in vitro fertilization (IVF) without ICSI. The report of treatment cycles by fertility centers is not uniformly required, and some cycles may be missed. Moreover, BORN started incorporating CARTR Plus data starting in January 2013. To maximize ascertainment of ART, we used BORN records on conception type to supplement CARTR Plus records.

### Confounders

Potential confounding variables accounted for were maternal age, parity, prepregnancy obesity (defined by prepregnancy body mass index), maternal smoking, social drug use or alcohol consumption, folic acid intake during pregnancy, mental health illness during prepregnancy or pregnancy (a composite measure of psychiatric disorders, depression, and anxiety), and prepregnancy physical health problems (a composite measure of chronic hypertension, diabetes, heart disease, thyroid disease, lupus, alcoholism, asthma, HIV infection, and hepatitis B). We also attempted to account for maternal educational level and family income. Because no individual records on maternal educational level and family income are available in the BORN Information System, we created 2 neighborhood-level variables for Ontario by converting postal codes affiliated with the mother into dissemination areas from the Canada census data nearest to the study period: (1) percentage of having a university degree among the adult population aged 25 to 64 years and (2) median family income, both measured in quintiles according to methods established in an earlier study.^35^ Dissemination areas were small, relatively stable geographic units with a population of 400 to 700 persons of relative homogenous socioeconomic status.^36^ We were careful in selecting confounders to be adjusted in the analysis so that no overadjustment would occur.^37,38^ All confounders included in the analysis were considered to be independently associated with CHD (outcome) and ART (exposure), and we verified the potential confounders by examining the association between confounders and outcome among unexposed study participants only.^39^

### Mediator

Twins have remarkably higher risks of adverse perinatal outcomes including CHD,^21,22,24,25,26^ and the proportion of twins in ART pregnancies is substantially higher than natural pregnancies.^1,2,19,20,21,22,23^ Because twin status is the intermediate step between ART and CHD (twin status is determined before the development of CHD), it qualified as a potential mediator.^40,41,42,43^

The pregnancy identification (an internal identifier assigned by BORN) was used to link records of the infant with the same pregnancy to obtain maternal information, and the birth identification (an internal identifier assigned by BORN) was used to link the records of the same infant across different periods within the BORN Information System. Unencrypted health card numbers were used to link BORN Information System records with CIHI records. The record linkage process is shown in the eFigure in the Supplement.

### Statistical Analysis

Statistical analysis was performed from January 1, 2017, to September 9, 2019. All analyses were performed using SAS, version 9.4 (SAS Institute Inc). We first compared baseline characteristics between ART and non-ART pregnancies and between the 2 subgroups of ART. We then described the prevalence of CHD as associated with potential confounding risk factors. Before multivariable regression analysis, multiple imputation using a fully conditional specification method^44,45,46^ was applied to create complete data sets. Details of missing imputation are provided in the eMethods in the Supplement. Multivariable logistic regression analyses were then performed in both exposure-outcome and mediator-outcome models while accounting for covariates. The variable ART was assessed as a binary variable. Because CHD is a rare outcome (<5%), we used odds ratios (ORs) to estimate relative risk in logistic regression models. All prespecified risk factors were included in the analysis.

To assess potential mediation of twinning in the association between ART and CHD, the Causalmed procedure^47^ in SAS was performed. The specific issue to be addressed by the mediation analysis was (1) how much of the overall association of ART with CHD risk was attributable to the association of ART with increased risk of twinning, which in turn affects CHD risk, and (2) how much of the association of ART and CHD did not involve twinning.^40,41,42,43^ The mediation analysis was done to parse the total association of ART with CHD prevalence into 2 components. The natural direct association of ART with CHD risk in the study population would occur if the pathway from ART to twin status were disabled. The Figure gives the contribution of the ART-CHD pathway to the overall association of ART with CHD risk. The natural indirect association, which is the association of ART with CHD risk in the study population, would occur if the direct ART-CHD pathway were disabled so that ART was associated with CHD risk only via the ART-twin-CHD pathway. The proportion mediated was defined as the natural indirect association divided by the total association, although it is only meaningful when both components of the total association have the same mathematical sign. Mediation analysis allows for control of confounding factors in estimating these results.

**Figure.**
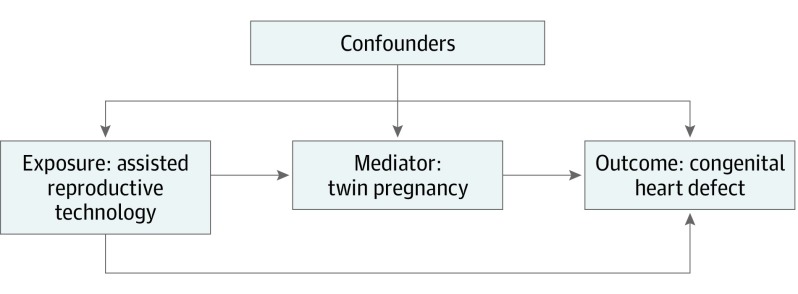
Framework of the Association Between Assisted Reproductive Technology and Congenital Heart Defects With Twin Pregnancy as the Mediator

Four-way decompositions of the total excess association were further estimated considering the potential interaction of ART with twinning in the association with risk of CHD using risk difference.^42^ The question of 4-way decompositions analysis is the following: To what extent is ART associated with CHD risk differently in twin pregnancies than in singleton pregnancies? The ART twin-status interaction can be operative regardless of the relative contributions of the direct and indirect paths (Figure). The ART twin-status interaction could be important for 2 reasons. First, it could help toward understanding the biologic mechanisms that underlie the association between ART and CHD. Why might ART affect twin pregnancies differently from how it affects singleton pregnancies in terms of CHD risk? Second, the amount of ART-twin interaction affects the degree to which results of mediation analysis obtained from this study are generalizable to other populations. The first of 4 components assessed in the 4-way decompositions was the controlled direct association, which was the CHD risk difference between ART and non-ART pregnancies among singleton births (the baseline risk difference in the absence of mediation or interaction). The second was reference interaction, which was the contribution of the ART-twin interaction if the frequency of twinning in ART pregnancies was set to what was observed among non-ART pregnancies (the association between CHD risk and the ART-twin interaction in the absence of mediation). The third component was the mediated interaction, which was the contribution of the ART-twin interaction when applied only to the added increase in the frequency of twinning seen in ART pregnancies (the combined association between CHD risk and mediation and the ART-twin interaction). The fourth component was the pure indirect association, which was the increase in CHD risk associated with the increased proportion of twins seen in ART pregnancies apart from the ART-twin interaction (mediation by twin status in the absence of an ART-twin interaction).

Preliminary analysis showed that there was no major difference between IVF and ICSI in terms of their association with CHD. To preserve the statistical precision and to simplify the interpretation, mediation analysis was performed for overall ART only. To assess robustness of the results, analyses deleting missing data without missing imputation and CHDs ascertained at birth or up to 1 year after birth (ie, without linking CHDs with fetal losses or terminations) were performed.

## Results

### Baseline Characteristics

A total of 507 390 mother-infant pairs with a singleton or twin birth were matched and included in final analysis. Among these, 10 149 (2.0%) were assisted pregnancies and 497 241 (98.0%) were nonassisted pregnancies. Women who conceived by ART were more likely to be older, to be experiencing their first pregnancy, and to have a higher body mass index. They were more likely to live in higher-income and higher-educational-level neighborhoods and have physical health problems but were less likely to have major mental health problems (such as schizophrenia or bipolar disease) and less likely to engage in smoking, social drug use, or alcohol consumption during pregnancy compared with women who conceived without ART (Table 1). The twin rate differed between groups: 18.5% (1881 of 10 149) in assisted pregnancies and 1.4% (7082 of 497 241) in nonassisted pregnancies. Women who conceived by ICSI were more likely to experience their first pregnancy (73.7% [2825 of 3835] vs 60.0% [3655 of 6094]) but less likely to be obese (16.8% [534 of 3187] vs 19.2% [945 of 4929]) than women who conceived by IVF. Twin rates were 17.5% in pregnancies conceived by ICSI and 19.2% in pregnancies conceived by IVF.

**Table 1.** Demographic Characteristics and Medical History of Women With ART and Non-ART Pregnancies^a^

Variable	ART Pregnancy			Non-ART Pregnancy (n = 497 241)
	ICSI (n = 3938)	IVF (n = 6211)	Total (n = 10 149)	
Maternal age at birth, mean (SD), y	35.7 (4.7)	35.8 (5.2)	35.8 (5.0)	30.3 (5.3)
Primaparous	2825 (73.7)	3655 (60.0)	6480 (65.3)	209 768 (42.9)
Obesity^b^	534 (16.8)	945 (19.2)	1479 (18.2)	77 034 (18.1)
Median family income at dissemination-area level, %				
>80	1179 (31.1)	1598 (27.2)	2777 (28.7)	77 548 (16.4)
60-80	1077 (28.4)	1661 (28.3)	2738 (28.3)	109 972 (23.2)
40-59	661 (17.4)	1076 (18.3)	1737 (18.0)	90 770 (19.1)
20-39	471 (12.4)	817 (13.9)	1288 (13.3)	85 745 (18.1)
<20	408 (10.8)	714 (12.2)	1122 (11.6)	110 344 (23.3)
University degree at dissemination-area level, %				
>80	1307 (34.2)	1763 (29.9)	3070 (31.6)	87 828 (18.4)
60-80	1135 (29.7)	1708 (28.9)	2843 (29.2)	114 984 (24.0)
40-59	665 (17.4)	1225 (20.7)	1890 (19.4)	104 229 (21.8)
20-39	465 (12.2)	751 (12.7)	1216 (12.5)	90 568 (18.9)
<20	246 (6.4)	460 (7.8)	706 (7.3)	80 656 (16.9)
Maternal				
Smoking	43 (1.1)	99 (1.6)	142 (1.4)	50 929 (10.2)
Alcohol consumption	43 (1.1)	57 (0.9)	100 (1.0)	9936 (2.0)
Social drug use	8 (0.2)	31 (0.5)	39 (0.4)	9962 (2.0)
Folic acid intake	2919 (74.1)	4536 (73.0)	7455 (73.5)	348 655 (70.1)
Mental health illness during prepregnancy or pregnancy				
All types	405 (10.3)	706 (11.4)	1111 (11.0)	68 778 (13.8)
Schizophrenia or bipolar disorders	10 (0.3)	16 (0.3)	26 (0.3)	2698 (0.5)
Depression	191 (4.9)	356 (5.7)	547 (5.4)	34 633 (7.0)
Anxiety	233 (5.9)	366 (5.9)	599 (5.9)	34 738 (7.0)
Prematernal health condition				
Any	1142 (29.0)	1770 (28.5)	2912 (28.7)	86 304 (17.4)
Chronic hypertension	74 (1.9)	112 (1.8)	186 (1.8)	4563 (0.9)
Diabetes, type 1 or type 2	37 (0.9)	91 (1.5)	128 (1.3)	4846 (1.0)
History of disease				
Heart	126 (3.2)	195 (3.1)	321 (3.2)	10 108 (2.0)
Pulmonary	108 (2.7)	213 (3.4)	321 (3.2)	19 570 (3.9)
Endocrine	584 (14.8)	856 (13.8)	1440 (14.2)	22 445 (4.5)
Twin birth	687 (17.5)	1194 (19.2)	1881 (18.5)	7082 (1.4)

Abbreviations: ART, assisted reproductive technology; ICSI, intracytoplasmic sperm injection; IVF, in vitro fertilization.

^a^ Data are presented as number (percentage) of women unless otherwise indicated. Only singleton or twin births were included in this cohort (N = 507 390); 71 records were excluded because of missing number of fetuses, and 185 records were excluded because the number of fetuses was more than 2. Percentages were calculated after missing values on individual variables were excluded from the group denominators showing on top of the table.

^b^ Obesity defined as body mass index of at least 30 (calculated as weight in kilograms divided by height in meters squared).

### Association of CHD With Potential Confounding Risk Factors

Table 2 shows the association of CHD found in infants with potential maternal confounding risk factors. The prevalence of CHD was higher among infants born to older mothers (adjusted OR, 1.40 [95% CI, 1.31-1.51] for women >35 years vs <30 years); lower-income mothers (adjusted OR, 1.10 [95% CI, 1.00-1.20] for women of family income at 40%-59% levels; 1.06 [95% CI, 0.96-1.16] at 20%-39% levels; and 1.12 [95% CI, 1.02-1.22] at <20% levels compared with women of family income >80% level); mothers residing in lower-educational-level neighborhoods (adjusted OR, 1.13 [95% CI, 1.04-1.24] for women residing in neighborhoods at 60%-80% levels of university degree; 1.19 [95% CI, 1.09-1.31] at 40%-59% levels; 1.27 [95% CI, 1.16-1.40] at 20%-39% levels; and 1.28 [95% CI, 1.16-1.41] at <20% levels compared with women residing in >80% level neighborhoods); those who smoked or used drugs or consumed alcohol (adjusted OR, 1.39 [95% CI, 1.30-1.50] compared with those who did not); those who did not receive folic acid during pregnancy (adjusted OR, 1.08 [95% CI, 1.02-1.14] compared with those who did); those who had major physical problems (adjusted OR, 1.77 [95% CI, 1.67-1.88] compared with those who did not); those who had major mental health problems (adjusted OR, 1.26 [95% CI, 1.18-1.35] compared with those who did not); and those with obesity (adjusted OR, 1.21 [95% CI, 1.13-1.30] compared with those who were nonobese).

**Table 2.** Association of Congenital Heart Defects With Potential Confounding Risk Factors Among Non-ART Pregnancies^a^

Exposure	Total No.	Congenital Heart Defects, No. (%)	OR (95% CI)	
			Crude	Adjusted
Maternal age, y				
<30	209 661	2491 (1.2)	1 [Reference]	1 [Reference]
30-34	205 291	2285 (1.1)	0.94 (0.88-0.99)	1.02 (0.96-1.08)
>35	82 198	1275 (1.6)	1.31 (1.22-1.4)	1.40 (1.31-1.51)
Missing data	91	6 (6.6)	NA	NA
Parity				
None	209 768	2454 (1.2)	0.93 (0.88-0.98)	0.99 (0.93-1.04)
≥1	279 673	3524 (1.3)	1 [Reference]	1 [Reference]
Missing data	7800	79 (1.0)	NA	NA
Family income at dissemination-area level, median, %				
>80	77 548	838 (1.1)	1 [Reference]	1 [Reference]
60-80	109 972	1190 (1.1)	1.00 (0.92-1.09)	0.96 (0.87-1.05)
40-59	90 770	1170 (1.3)	1.20 (1.09-1.31)	1.10 (1.00-1.20)
20-39	85 745	1092 (1.3)	1.18 (1.08-1.29)	1.06 (0.96-1.16)
<20	110 344	1508 (1.4)	1.27 (1.17-1.38)	1.12 (1.02-1.22)
Missing data	22 862	259 (1.1)	NA	NA
University degree at dissemination-area level, %				
>80	87 828	877 (1.0)	1 [Reference]	1 [Reference]
60-80	114 984	1304 (1.1)	1.14 (1.04-1.24)	1.13 (1.04-1.24)
40-59	104 229	1281 (1.2)	1.23 (1.13-1.34)	1.19 (1.09-1.31)
20-39	90 568	1233 (1.4)	1.37 (1.25-1.49)	1.27 (1.16-1.40)
<20	80 656	1163 (1.4)	1.45 (1.33-1.58)	1.28 (1.16-1.41)
Missing data	18 976	199 (1.1)	NA	NA
Maternal smoking, social drug use, or alcohol consumption				
Yes	60 310	1081 (1.8)	1.58 (1.48-1.69)	1.39 (1.30-1.50)
No	436 931	4976 (1.1)	1 [Reference]	1 [Reference]
Folic acid intake during pregnancy				
Yes	348 655	4186 (1.2)	1 [Reference]	1 [Reference]
No	148 586	1871 (1.3)	1.05 (0.99-1.11)	1.08 (1.02-1.14)
All types of mental health illness during prepregnancy or pregnancy				
Yes	68 778	1181 (1.7)	1.52 (1.42-1.62)	1.26 (1.18-1.35)
No	428 463	4876 (1.1)	1 [Reference]	1 [Reference]
All types of prepregnancy maternal health conditions				
Yes	86 304	1716 (2.0)	1.90 (1.80-2.01)	1.77 (1.67-1.88)
No	410 937	4341 (1.1)	1 [Reference]	1 [Reference]
Maternal prepregnancy obesity, body mass index^b^				
Obese	77 034	1196 (1.6)	1.37 (1.28-1.46)	1.21 (1.13-1.30)
Nonobese	348 386	3973 (1.1)	1 [Reference]	1 [Reference]
Missing data	71 821	888 (1.2)	NA	NA

Abbreviations: ART, assisted reproductive technology; NA, not applicable; OR, odds ratio.

^a^ Adjusted ORs and 95% CIs were generated from 1 logistic regression model adjusting for all independent variables listed in the table simultaneously.

^b^ Obesity defined as body mass index of at least 30 (calculated as weight in kilograms divided by height in meters squared).

### Association of CHD With ART and Twin Pregnancies

Table 3 displays the results of the analysis of the association of CHD with ART and twinning and ART-twin interactions. Infants born to mothers who conceived by ART had a higher prevalence of CHD than did infants born to mothers who conceived without using ART (2.2% [223 of 10 149] vs 1.2% [6057 of 497 241]; crude OR, 1.82; 95% CI, 1.59-2.09). This association decreased after adjusting for several risk factors simultaneously. For specific ART techniques, infants born to mothers who conceived by ICSI had higher risk of CHD than did infants born to mothers who conceived by IVF (2.3% vs 2.1%). Twins had a higher prevalence of CHD than singletons (5.5% vs 1.2%; OR, 4.91; 95% CI, 4.47-5.40). This association decreased after adjusting for several risk factors simultaneously. Prevalence of CHD was the highest in non-ART twin pregnancies (5.6%), second highest in ART twin pregnancies (5.1%), second lowest in ART singleton pregnancies (1.6%), and lowest in non-ART singleton pregnancies (1.2%).

**Table 3.** Association of Congenital Heart Defects With ART and Twin Status

Exposure Status	Congenital Heart Defects, No. (%)	Risk Difference vs Reference	OR (95% CI)	
			Crude	Adjusted
**Main** A**ssociations**^**a**^				
Intracytoplasmic sperm injection (n = 3938)	91 (2.3)	1.1	1.92 (1.56-2.37)	1.82 (1.47-2.25)
In vitro fertilization (n = 6211)	132 (2.1)	0.9	1.76 (1.48-2.10)	1.63 (1.36-1.94)
Overall ART (n = 10 149)	223 (2.2)	1.0	1.82 (1.59-2.09)	1.70 (1.48-1.95)
Non-ART pregnancies (n = 497 241)^b^	6057 (1.2)	[Reference]	1 [Reference]	1 [Reference]
Twins (n = 8963)	489 (5.5)	4.3	4.91 (4.47-5.40)	4.69 (4.24-5.18)
Singletons (n = 498 427)	5791 (1.2)	[Reference]	1 [Reference]	1 [Reference]
**ART-Twin Interaction**^b^				
ART pregnancies				
Twin (n = 1881)	95 (5.1)	3.9	4.55 (3.70-5.60)	4.29 (3.47-5.30)
Singleton (n = 8268)	128 (1.6)	0.4	1.35 (1.13-1.61)	1.26 (1.05-1.51)
Non-ART pregnancies				
Twin (n = 7082)	394 (5.6)	4.4	5.04 (4.54-5.60)	4.93 (4.43-5.47)
Singleton (n = 490 159)	5663 (1.2)	[Reference]	1 [Reference]	1 [Reference]

Abbreviations: ART, assisted reproductive technology; OR, odds ratio.

^a^ Adjusted covariates included maternal age; parity; quintiles of median family income at the dissemination-area level; quintile of percentage of university degree at the dissemination-area level; prepregnancy obesity; folic acid intake during pregnancy; maternal smoking, social drug use, and alcohol consumption; mental health illness during prepregnancy or pregnancy; and prepregnancy health condition and obesity status.

^b^ Adjusted for ART in addition to all covariates listed above.

### Mediation Analysis

Table 4 presents results of mediation analysis with twinning acting as the mediator between ART and CHD. The results showed that the association between ART and CHD was largely mediated by twinning (adjusted OR, 1.68; 95% CI, 1.44-1.92). The OR of the natural direct association of ART with CHD among singleton pregnancies was 1.09 (95% CI, 0.93-1.25).

**Table 4.** Analysis of ART and Congenital Heart Defects Association With Twin Pregnancy as a Mediator^a^

Mediation Association	Estimate, Odds Ratio (95% CI)
Total	1.68 (1.44-1.92)
Natural direct	1.09 (0.93-1.25)
Natural indirect	1.55 (1.47-1.62)

Abbreviation: ART, assisted reproductive technology.

^a^ The proportion mediated was 87.3%. Exposure variable: ART; mediator: twin pregnancy; interaction: ART × twin pregnancy; and covariates: maternal age; parity; quintiles of median family income at the dissemination-area level; quintile of percentage of university degree at the dissemination-area level; prepregnancy obesity; maternal smoking, social drug use, or alcohol consumption; folic acid intake during pregnancy; all types of mental health illness during prepregnancy or pregnancy; and all types of prepregnancy maternal health conditions.

The results of 4-way decompositions analysis found that reference interaction and mediated interaction together accounted for 7.9% of the total association, and neither component was statistically significantly different from 0. Mediation through twinning accounted for more than 80% of the excess association (eTable 2 in the Supplement). Results of multiple logistical regression analysis deleting missing data or restricting to pregnancy outcome yielded similar results in terms of direction and magnitude of the association of CHD with ART and twinning (eTable 3 in the Supplement).

## Discussion

This large population-based study found that compared with infants born to mothers who conceived without using ART, infants born to mothers who conceived with ART were at higher risk of CHD. The association between ART and CHD was largely mediated by twinning. The contributions to overall excess risk from other associations of ART (not involving twinning) and from the ART-twin interaction were smaller and not statistically significant. However, the results do not rule out possible small contributions of those mechanisms to excess risk.

Our mediation analysis is different from previous studies of the contribution of twinning in the literature. Previous studies^40,41,42,43^ have used different strategies to analyze the association of ART, twinning, and CHD, with most considering twinning as a confounder. Treating a mediator as a confounder in an analysis is flawed.^40,41,42,43^ Mediation analysis helped to assess the association between ART (exposure) and CHD (outcome). We believe that ignoring twinning as a mediator would lead to unexplainable or paradoxical findings. The finding that twinning contributed up to 87% of the association between ART and CHD may be important in the clinical practice of perinatal medicine. For example, instead of performing fetal echocardiography in ART pregnancies,^32^ it may be more cost-efficient to perform this procedure in twin pregnancies. Policy makers may consider a policy of single embryo transfer. Since 2015, Ontario government-provided public funding for IVF treatment required single embryo transfer in most women younger than 38 years.^48^ Some other adverse perinatal outcomes may share mechanisms of CHD,^49^ and a similar analytic strategy could be applied in future studies to assess these associations as well.

### Strengths and Limitations

Our study has several strengths. First, the study sample was based on the entire population from 1 Canadian province (Ontario). Because BORN has 100% capture for hospital and home births in Ontario, our study was free from bias caused by selective inclusion of births. Second, the quality of BORN data has been evaluated by a number of studies,^50^ and it has been considered to be high. Third, the sample size of our study was the largest reported to date in this field. Fourth, we used multiple sources to identify CHD, including birth records, pregnancies that were lost or terminated after prenatal screening and in antenatal clinics (a total of 198 CHD cases were ascertained from losses or terminations), and cases diagnosed after birth (≤1 year) through record linkage with CIHI databases, with better ascertainment of outcome. Ascertainment of CHD cases in aborted fetuses might be more complete in ART pregnancies than in non-ART pregnancies, resulting in an artificially higher prevalence of CHD after ART. To address this possibility, we performed a sensitivity analysis restricted to pregnancy outcomes. The result of this sensitivity analysis was similar to the main results (eTable 3 in the Supplement), suggesting that ascertainment bias is not a serious threat to the validity of our study results. Fifth, rich demographic and clinical information collected by BORN allowed a thorough adjustment for confounding, assessment of mediation, and subgroup analyses. The use of mediation analysis helped to sort the associations of ART with twinning and CHD. Although the data contain no information on infertility, some important risk factors that are associated with both infertility and CHD have been adjusted. As a result, we were able to control some of the confounding by infertility, although not completely.

Several limitations of our study should be recognized. First, some important variables, such as ethnicity, household income, and maternal education, were either not available or not measured at an individual level. Second, the higher socioeconomic status and healthy behaviors of couples who underwent ART compared with couples who conceived without ART may confound the observed results. Although we adjusted several maternal characteristics to mitigate confounding, administrative data cannot guarantee complete and accurate data collection. As a result, residual confounding may still exist. As VanderWeele and Ding^50^ suggested, a central concern about observational data are unmeasured or uncontrolled confounding, and with observational data, we can never be certain that the adjustments are adequate.^51^ Third, we were not able to address the issue of donor gametes, which may have a different association with the outcome for ART pregnancies compared with own gametes.^1^ Fourth, there were missing values for variables included in the regression analysis. We used multiple imputation to create a complete data set to mitigate the consequences of missing values in the analysis. However, analysis deleting records with missing variables or deleting CHDs ascertained from fetal losses or termination yielded similar results, suggesting the robustness of the data used in this study. Fifth, if the ART-twin interaction was present, both the natural indirect association and the natural direct association depended on the population frequency of twinning.^43^ The ART-twin interaction was small and not clinically significant, whereas the difference between the natural direct association and the natural indirect association was large and clinically significant, and the interpretation of the natural direct association and the natural indirect association was unaffected by the ART-twin interaction. Sixth, we were not able to address zygosity and chronicity of twins. Because the main focus of this study was the indirect association of twinning with CHD and ART, and zygosity and chronicity of twins may be a lesser concern in the assessment of indirect association than an assessment of direct association. Seventh, because CARTR Plus data on IVF cycles is not completely captured by BORN, we used information from BORN to supplement the ascertainment of ART. As a result, some ICSI cases in BORN may have been misclassified as IVF because BORN data sometimes lack details to differentiate ICSI from IVF. Because the focus of this study was an ART and non-ART comparison, it may be acceptable to have some IVF or ICSI misclassification to ensure a complete ascertainment of ART.

## Conclusions

This study found that the observed association between ART and CHD may be substantially mediated by twinning. We believe that this finding is important to the clinical practice of perinatal medicine and to policy making. The robustness of this analytic strategy should be tested in the assessment of the association of ART with CHD in other populations and ART with other perinatal outcomes that may share similar mechanisms of CHD.

## Notes

Supplement. **eMethods.** Description of Missing Imputation Process **eTable 1.** *ICD-10-CA* Diagnosis Code for CHD in CIHI DAD and NACRS Databases **eTable 2.** Four-way Decompositions of Risk Difference **eTable 3.** Results of Sensitivity Analysis for the Association of ART with CHD, Ontario, Canada, April 1, 2012, and October 31, 2015 **eFigure.** Record Linkage Strategy for the Study of the Association between ART and CHD, Ontario, Canada, April 1, 2012, to October 31, 2015
