# Supplementary material for: Associations of Assisted Reproductive Technology and Twin Pregnancy With Risk of Congenital Heart Defects
Source: JAMA Pediatr. 2020 Feb 24;174(5):1–9. doi: 10.1001/jamapediatrics.2019.6096 (PMC7042937; doi:10.1001/jamapediatrics.2019.6096)
Supplement: Supplement. — eMethods. Description of Missing Imputation Process eTable 1. ICD-10-CA Diagnosis Code for CHD in CIHI DAD and NACRS Databases eTable 2. Four-way Decompositions of Risk Difference eTable 3. Results of Sensitivity Analysis for the Association of ART with CHD, Ontario, Canada, April 1, 2012, and October 31, 2015 eFigure. Record Linkage Strategy for the Study of the Association between ART and CHD, Ontario, Canada, April 1, 2012, to October 31, 2015 [file jamapediatr-174-446-s001.pdf]

Supplementary Online Content

Wen SW, Miao Q, Taljaard M, et al. Associations of assisted reproductive technology and twin pregnancy with risk of congenital heart defects. *JAMA Pediatr*. Published online February 24, 2020. doi:10.1001/jamapediatrics.2019.6096

eMethods. Description of Missing Imputation Process

eTable 1. *ICD-10-CA* Diagnosis Code for CHD in CIHI DAD and NACRS Databases

eTable 2. Four-way Decompositions of Risk Difference

eTable 3. Results of Sensitivity Analysis for the Association of ART with CHD, Ontario, Canada, April 1, 2012, and October 31, 2015

eFigure. Record Linkage Strategy for the Study of the Association between ART and CHD, Ontario, Canada, April 1, 2012, to October 31, 2015

© 2020 Wen SW et al. *JAMA Pediatrics.*

eMethods. Description of Missing Imputation Process

The fully conditional specification method uses a sequential regression procedure to impute missing values, assuming a joint distribution for all variables under the assumption of Missing-At-Random (1, 2). A linear regression imputation model was used for maternal age and BMI, while a generalized logit model was used for household income, education level, and parity. All variables used in multivariable analysis were included in imputation models. A total of five imputed datasets were created. Results from the five imputation-completed datasets were combined using the rules developed by Rubin to account for imputation uncertainty (3).

References

1. van Buuren, S. Multiple Imputation of Discrete and Continuous Data by Fully Conditional Specification. Statistical Methods in Medical Research 2007;16:219–42.
2. Little RJA. & Rubin DB. Statistical Analysis with Missing Data, Second Edition, New York: John Wiley & Sons, 2002.
3. Rubin DB. Multiple Imputation for Nonresponse in Surveys. John Wiley & Sons Inc., New York, 2004.

© 2020 Wen SW et al. *JAMA Pediatrics.*

eTable 1. *ICD-10-CA* Diagnosis Code for CHD in CIHI DAD and NACRS Databases

Q20:	Congenital malformations of cardiac chambers and connections
Q21:	Congenital malformations of cardiac septa
Q22:	Congenital malformations of pulmonary and tricuspid valves
Q23:	Congenital malformations of aortic and mitral valves
Q24:	Other congenital malformations of heart<br>Include Q24.0, Q24.8, Q24.9,Q26.8,Q26.9, and Q24.2 to Q24.5;<br>Exclude Q24.1 (Laevocardia) andQ24.6 Congenital heart block
Q25:	Congenital malformations of great arteries<br>Include Q25.1 to Q25.9<br>Exclude Q25.0 (Patent ductus arteriosus
Q26:	Congenital malformations of great veins<br>Include Q26.0 to Q26.4<br>Q26.0 Congenital stenosis of vena cava<br>Q26.1 Persistent left superior vena cava<br>Q26.2 Total anomalous pulmonary venous connection<br>Q26.3 Partial anomalous pulmonary venous connection<br>Q26.4 Anomalous pulmonary venous connection, unspecified

© 2020 Wen SW et al. *JAMA Pediatrics.*

eTable 2. Four-way Decompositions of Risk Difference*

Four-way decompositions of total effect	Estimate	% of contribution in total risk difference
Controlled direct effect	0.08	11.76
Reference interaction	0.004	0.59
Mediated interaction	0.05	7.35
Pure indirect effect	0.55	80.88
Total effect	0.68	100

* Exposure variable: overall ART vs. natural pregnancy. Mediator: twin pregnancy.
Interaction: interaction between twin pregnancy and overall ART on the outcome of CHD.

© 2020 Wen SW et al. *JAMA Pediatrics.*

eTable 3. Results of Sensitivity Analysis for the Association of ART with CHD, Ontario, Canada, April 1, 2012, and October 31, 2015

	**Exposure status**	**N of CHD**	**% of CHD**	**Crude OR (95% CI)**	**Adjusted OR (95% CI) ***
Without missing imputation	ICSI (N=3,938)	91	2.31	1.92 (1.56-2.37)	1.69 (1.32-2.17)
	IVF (N=6,211)	132	2.13	1.76 (1.48-2.10)	1.76 (1.44-2.14)
	Overall ART N=10,149)	223	2.2	1.82 (1.59-2.09)	1.73 (1.48-2.03)
	Natural pregnancies (N=497,241)	6057	1.22	Reference	Reference
	Twins (N=8,963)	489	5.46	4.91 (4.47-5.40)	4.67 (4.17-5.24)
	Singletons (N= 498,427)	5791	1.16	Reference	Reference
Included only CHD cases ascertained at or after birth	ICSI (N=3,896)	89	2.28	1.95 (1.57-2.40)	1.70 (1.32-2.19)
	IVF (N=6,146)	128	2.08	1.77 (1.48-2.11)	1.75 (1.43-2.14)
	Overall ART (N= 10,042)	217	2.16	1.84 (1.60-2.11)	1.73 (1.48-2.03)
	Natural pregnancies (N= 493,764)	5865	1.19	Reference	Reference
	Twins (N=8,867)	486	5.48	5.07 (4.61-5.58)	4.82 (4.30-5.41)
	Singletons (N= 494,939)	5596	1.13	Reference	Reference

* For ART and type of ART: adjusted covariates included maternal age, parity, quintiles of median family income on dissemination area level, quintile of percentage of university degree on dissemination area level, pre-pregnancy obesity, folic acid intake during pregnancy, maternal smoking or social drug use or alcohol consumption, mental health illness in pre-pregnancy or during pregnancy, and pre-pregnancy maternal health condition. In addition, for the exposure of twins, overall ART was also adjusted in the model.

© 2020 Wen SW et al. *JAMA Pediatrics.*

eFigure. Record Linkage Strategy for the Study of the Association Between ART and CHD, Ontario, Canada, April 1, 2012, to October 31, 2015

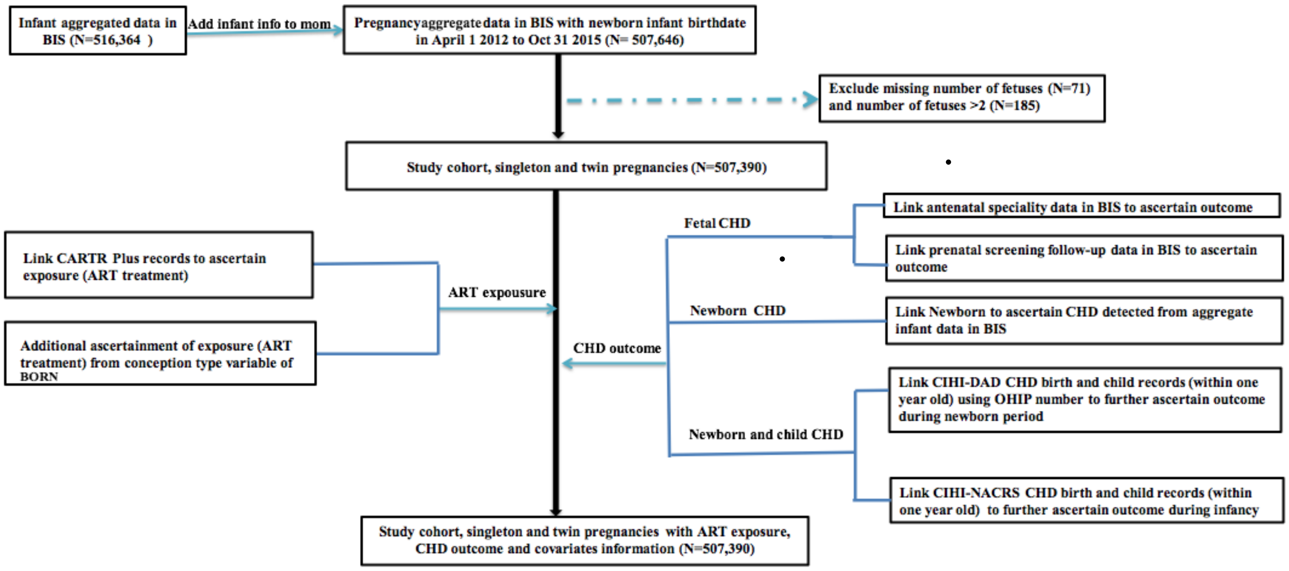

© 2020 Wen SW et al. *JAMA Pediatrics.*
